# Optimization of Cold Brew Coffee Using Central Composite Design and Its Properties Compared with Hot Brew Coffee

**DOI:** 10.3390/foods12122412

**Published:** 2023-06-19

**Authors:** Nur Hadiyani Zakaria, Kanyawee Whanmek, Sirinapa Thangsiri, Wimonphan Chathiran, Warangkana Srichamnong, Uthaiwan Suttisansanee, Chalat Santivarangkna

**Affiliations:** Institute of Nutrition, Mahidol University, Salaya, Phuttamonthon, Nakhon Pathom 73170, Thailand; hadiyanizakaria@gmail.com (N.H.Z.); kanyaweebiosci@gmail.com (K.W.); sirinapa.tha@mahidol.ac.th (S.T.); wimonphan.cha@mahidol.ac.th (W.C.); warangkana.sri@mahidol.ac.th (W.S.); uthaiwan.sut@mahidol.ac.th (U.S.)

**Keywords:** cold extraction, response surface methodology, caffeine content, antioxidant activity, organic acid

## Abstract

The cold brew coffee (CBC) trend is increasing globally; nevertheless, there is limited literature on this popular beverage. Many studies have focused on the health benefits of green coffee beans and coffee brewed by conventional hot water methods. Thus, whether cold brew conveys similar benefits is still unclear. This study aimed to investigate the influences of brewing conditions on physicochemical properties using response surface methodology in order to optimize the brewing parameters and compare the resulting CBC with the coffee from the French Press method. Central Composite Design was used to evaluate the effects and optimize the brewing parameters (i.e., water temperature, coffee-to-water ratio (C2WR), coffee mesh size, and extraction time) on total dissolved solids (TDS). Physicochemical properties, antioxidant activity, volatile compounds, and organic acids were compared between CBC and its French Press counterpart. Our results showed that water temperature, C2WR, and coffee mesh size significantly influenced the TDS of CBC. The optimized brewing conditions were water temperature (4 °C), C2WR (1:14), coffee mesh size (0.71 mm), and 24-h extraction time. At similar TDS, caffeine content, volatile compounds, and organic acids were higher in CBC, while other properties showed no significant difference. In conclusion, this study showed that at similar TDS, CBC has characteristics generally similar to hot brew coffee, except for the caffeine and sensory-related compound content. The model for the prediction of TDS from this study may benefit food services or industries for the optimization of brewing conditions to obtain different characteristics of CBC.

## 1. Introduction

Globally, coffee is one of the most widely consumed beverages, with reported consumption of approximately 166.63 million coffee bags (60 kg each) between 2020 and 2021 [1]. Drinking coffee provides not only pleasure and experience but also health benefits [2]. Studies have been conducted on coffee and reported that coffee contains over 1000 bioactive compounds with antioxidant, anti-inflammatory, and anti-cancerous properties [3]. Many studies have linked coffee consumption with a lower risk of all-cause mortality, cardiovascular mortality, cancer, metabolic, neurologic, and liver disease [3]. However, most studies on the health benefits of coffee have been conducted on either green coffee or conventional coffee brewing methods. Often, the methods used were not clearly described. Cold brew coffee (CBC), which is gaining interest due to its popularity among consumers, has been claimed to have health benefits superior to its hot brew counterpart [4]. There is limited scientific literature to support this claim, which leaves a gap in determining whether cold extraction renders distinct benefits besides the organoleptic properties.

There are various ways to make coffee [5], but due to the increasing popularity of cold brew coffee (CBC), the global cold brew market is expected to reach $1.63 billion by 2025, up from $420.9 million in 2019 [6]. In contrast to the market growth of CBC, there is currently no guideline or recommendation for the method of CBC preparation, whereas the definition by Fuller and Rao (2017) is well accepted and used by various researchers [7,8,9,10]. The research on CBC brewing conditions is limited, and many works investigate two or three brewing variables without taking the other interdependent variables into account. Furthermore, the scientific literature on the parameters affecting the characteristics of CBC was limited and contradictory. This provides room for other researchers to adjust brewing variables that influence the final brew, i.e., water temperature, C2WR, extraction time, and coffee grind particle.

A perfect cup of coffee can be evaluated by its sensory, nutritional, or physicochemical characteristics [11]. Nutritional and physicochemical properties can be measured objectively. Even though sensory experience, perception, and consumer preference are subjective parameters, they are the main factors in determining the quality of a beverage [5]. These sensory experiences and preferences vary among individuals due to their geographical, cultural, and social context [12]. In order to have a more objective and quantifiable parameter to evaluate the quality of coffee, we can measure certain physicochemical characteristics like total dissolved solids (TDS) and the extraction yield of the coffee [5]. The Specialty Coffee Association of America has developed the golden cup standard for hot brew coffee and states that an ideal coffee should have a TDS ranging between 1.15 and 1.35% with an extraction yield between 18 and 22% [13]. While the brewing practices for hot brew have been established, those for cold brew remain unclear due to a lack of scientific literature. Therefore, the objective of this study was to optimize CBC conditions and compare its physicochemical and chemical properties with those of its hot counterpart from the French Press method.

## 2. Materials and Methods

### 2.1. Sample Collection and Preparation

Thai Blue Mountain coffee beans (10 kg) were purchased from Linlin Coffee Equipment (Bangkok, Thailand) and packaged into 500 g aluminum laminated bags fitted with one-way valves and heat sealed. The beans were stored at −20 °C and brought out of storage to bring them to room temperature before grinding them into coffee powder.

The coffee beans were ground using the Severin 100 W KM 3874 coffee grinder (Sundern, Germany) at setting numbers ranging from 1 and 9 and passed through the Retsch Type Vibro vibratory sieve shaker (Haan, Germany). The coffee grinds were sieved to mesh size # 25 (0.71 mm), # 35 mesh (0.50 mm), and # 60 mesh (0.25 mm). The ground coffee samples were packaged into aluminum pouches, vacuum sealed, and stored at −20 °C according to their sieved mesh sizes. One hour prior to coffee brewing, the coffee grinds were taken out of storage to bring them to room temperature.

### 2.2. Cold Extraction via Full Immersion Method

The brewing parameters followed the experimental design for the central composite design setup. For the comparison between cold and hot brew coffee characteristics, the CBC was prepared using the optimized brewing conditions generated by Design of Expert software v13 (Minneapolis, MN, USA).

### 2.3. Hot Extraction via the French Press Method

Coffee was brewed using the method provided by the Specialty Coffee Association of America for French Press coffee [14]. Briefly, 36 g of ground coffee (mesh size # 35) was weighed and placed in the French Press coffee pot. Then, 540 mL of hot water (93 ± 1 °C) was added to the pot to saturate the coffee. After 2 min, the coffee was stirred, and the remaining 120 mL of hot water was poured into the pot. After 4 min, the plunger was slowly pressed down to the bottom of the pot. The brewed coffee was decanted, cooled, and stored at −20 °C before being analyzed. The coffee was brewed in three batches.

### 2.4. Determining Brewing Conditions for Cold Extraction through Central Composite Design

A three-level CCD was utilized to optimize the brewing conditions: water temperature, coffee mesh size, C2WR, and extraction time of CBC towards TDS. The design model was quadratic and fitted to characterize the nature of the response surface in the favored experimental region. Table 1 lists the effects of each parameter on the response being studied, which were at three different levels by combining two factorial points, two axial points, and a sole central point (+1, 0, −1). The TDS was the response observed. The experiment response was fitted to a second-order polynomial regression model, including significant linear, pairwise, and quadratic interaction coefficients to predict the optimal condition. The quadratic mathematical model used is shown in Equation (1):

(1) $${y=\beta }_{0}+\sum_{i=1}^{k}\beta_{i}x_{i}+\sum_{i=1}^{k}\beta_{ii}x_{i}^{2}+\sum_{1=i\leq j}^{k}\beta_{ij}{x_{i}x}_{j,\;i\neq j}$$

where y is the response variable, x is the independent factor that influences Y, β_0_ is the intercept, k is the number of involved factors, β_i_ is the ith linear coefficient, β_ii_ is the quadratic coefficient, β_ij_ the coefficient of interaction effect when i < j, with i and j = 1, 2, 3, and i ≠ j. All experiments were conducted in triplicate.

### 2.5. Physicochemical Analysis

#### 2.5.1. Total Dissolved Solids and Extraction Yield

The TDS were measured using a refractometer (Milwaukee Inc., Brookfield, WI, USA) with automatic temperature compensation. The refractometric readings were previously determined and found to be proportional to their TDS with a coefficient of 0.85, and the value was used to convert Brix to TDS. The results from the refractometer were represented as Brix (%) and calculated using Equation (2) [15].

Total Dissolved Solids (%) = Brix (%) × 0.85(2)

Utilizing the TDS, the extraction yield can be calculated yield using Equation (3) [15],

(3) $$\mathrm{Extraction}\;\mathrm{Yield}\;(\%)=\frac{TDS\times Brewed\;Coffee\;(g)}{Ground\;Coffee\;(g)}$$

#### 2.5.2. pH and Titratable Acidity

The pH and titratable acidity of coffee samples were determined using the method from Fuller and Rao (2018) [16], with slight modifications. The pH of each brewed coffee sample was measured with an Ohaus Starter 3100 bench pH meter, ST3100-B (Parsippany, NJ, USA). A 20 mL aliquot of coffee brew was titrated with NaOH (0.1N) at 25 °C to a pH of 6.0, 8.0, and 8.2. Results were expressed as the volume of NaOH per 20 mL of coffee.

### 2.6. Chemical Analysis

#### 2.6.1. High-Performance Liquid Chromatography (HPLC)

The organic acid profile of the coffee samples was determined by high-performance liquid chromatography using the method published by Luu et al. (2023) [17]. Briefly, the brewed coffee samples were filtered through a 0.22 µm nylon syringe filter. The filtrate (10 µL) was injected into a 5 µm Thermo Scientific HyperSil Gold aQ column (250 × 4.6 mm) (Thermo Fisher Scientific, Bremen, Germany) attached to a Waters Alliance HPL e2695 with a diode array detector (Waters Corporation, Milford, MA, USA). An isocratic solvent system comprising 50 mM phosphate buffer at pH 8.2 with a flow rate of 0.70 mL/min was set up. The total run time was 20 min, while the separation of organic acids was visualized at 214 nm (UV detection). The standards for organic acids used were HPLC-grade. Acetic acid was purchased from Merck (Darmstadt, Germany). Oxalic acid, malic acid, citric acid, succinic acid, and tartaric acid were purchased from Megazyme (Wicklow, Ireland). While formic acid, ascorbic acid, and propionic acid were bought from Sigma Aldrich (St. Louis, MO, USA). Fumaric acid and quinic acid were bought from Tokyo Chemical Industry (Portland, OR, USA). The HPLC chromatograms of the standards and samples are shown in Supplementary Figure S1.

#### 2.6.2. Liquid Chromatography-Electrospray Ionization Tandem Mass Spectrometry (LC-ESI-MS/MS)

The phenolic compounds and caffeine content of cold and hot brew coffee were determined using LC-ESI-MS/MS according to the method by Sirichai et al. (2022) [18]. The MS parameters were: mass range, 50–1000 *m*/*z*; positive ion, 3500 V; negative ion, 3500 V; sheath gas (N2), 30 Arb; auxiliary gas (N2), 15 Arb; ion transfer tube temperature, 325 °C; vaporizer temperature, 350 °C. The characterization of phenolics was conducted using data obtained with the selective reaction monitoring (SRM) mode.

The LC–ESI-MS/MS with a Chromeleon 7 chromatography data system (version 7.2.9.11323 from Thermo Fisher Scientific, Bremen, Germany) was used for molecular mass analysis of authentic standards of phenolics. The mixed solution of all authentic phenolics was prepared in methanol at different concentrations for calibration curves. The chromatographic separation of the standards was conducted on an Accucore RP-MS column (2.1 mm, 100 mm, 2.6 µm, Thermo Fisher Scientific, Bremen, Germany) by eluting with a mobile phase containing acetonitrile (eluent A) and 0.1% (*v*/*v*) formic acid (eluent B). The gradient elution at 10% A and 90% B was carried out at a flow rate of 0.5 mL/min for 10 min. The injection volume was 10 µL. The column temperature was maintained at 35 °C.

The validation of phenolics was described by Sirichai et al. (2022) [18]. The validations of caffeine are shown in Supplementary Tables S1 and S2. Three SRM transitions of caffeine at specific collision energies (V) and radio frequencies (RF-lens; V) were used to analyze the selectivity and specificity of caffeine detection in coffee samples. The caffeine standard calibration curve was prepared using standard stock solutions in methanol. The caffeine calibration curve range was carried out in triplicate. The limit of detection (LOD) and limit of quantification (LOQ) were evaluated from the standard curve according to Equations (4) and (5):

LOD = (3.3σ)/S (4)

LOQ = (10σ)/S (5)

where σ is the standard deviation of the Y-intercept, and S is the average slope of the linear calibration curve. Precision was determined using values obtained from seven replicate injections within the same day. The variations in retention time were presented as a percentage of the relative standard deviation (%RSD). Accuracy was established using a known amount of the caffeine standard. The solutions of the spiked samples were injected into the LC-ESI-MS/MS system with the SRM system in triplicates. The accuracy of this method was expressed as percentage recovery (% recovery) using Equation (6):

% recovery = [(observed amount − initial amount) × 100]/spiked amount (6)

Prior to analyzing the phenolic and caffeine content, a brewed coffee sample (1.5 L) was freeze-dried. The powder samples (0.5 g dry weight) were dissolved in a mixture of formic acid (40 mL) and 62.5% (*v*/*v*) methanol containing 0.5 g tert-butylhydroquinone (TBHQ) (10 mL) before shaking in a water bath shaker at 80 °C for 2 h (TW20 series from Julabo GmbH, Seelbach, Germany). The mixture was placed on ice for 5 min, added with 1% (*v*/*v*) ascorbic acid (100 µL), and sonicated for 5 min in an ultrasonic bath (Branson Ultrasonics™ M series, Branson Ultrasonics Corp., Danbury, CT, USA). The mixture was adjusted to 50 mL with 62.5% (*v*/*v*) methanol containing 0.5 g TBHQ and filtered through a PTFE membrane (0.22 µM).

Phenolic acid standards: chlorogenic acid, caffeic acid, syringic acid, p-coumaric acid, ferulic acid, sinapic acid, cinnamic acid, 4-hydroxybenzoic acid, and 3,4-dihydroxybenzoic acid were from Sigma-Aldrich. Gallic acid, vanillic acid, and rosmarinic acid were from the Tokyo Chemical Industry (Tokyo, Japan). Flavonoid standards: apigenin, (-)-epigallocatechin gallate, kaempferol, genistein, hesperidin, myricetin, luteolin, quercetin, and naringenin were from the Tokyo Chemical Industry. Isorhamnetin was from Extrasynthese (Genay, France). Galangin was from Wuhan ChemFaces Biochemical Co., Ltd. (Wuhan, China). Rutin was from Sigma-Aldrich. The chromatograms of phenolics and caffeine and the samples are shown in Supplementary Figures S2 and S3.

#### 2.6.3. Solid Phase Microextraction-Gas Chromatography/Mass Spectrometry (SPME-GC/MS)

Volatile compound analysis was conducted using SPME-GC/MS based on the method by Heo et al. [10] and Kang et al. [19] with slight modifications. Solid phase microextraction was achieved using SPME Fiber 50/30 µm Divinylbenzen/Carboxen/Polydimethylisoxane (DVB/CAR/PDMS) fiber assembly, needle size 24 Ga, Stableflex (Sigma-Aldrich). The total volume of the coffee sample in the vial was adjusted to 3 mL. The sample vials were conditioned for 15 min at 40 °C, and then the fiber was exposed for 10 min before injection. The fiber was injected and held for 30 s at 260 °C for the desorption of volatiles. The chromatographic separation was performed using an Agilent 7890A with a 5975C detector. The data were processed and analyzed using GCMSD software and the NIST library database (Agilent Technologies, Santa Clara, CA, USA). The programmed temperature was as follows: it started at 40 °C for 5 min and increased to 250 °C at a rate of 4 °C/min. The DB wax column (30 m × 0.25 mm, thickness 0.25 µm) (Agilent Technologies, Santa Clara, CA, USA) was used for the chromatographic separation, and the splitless injection mode was used. Helium gas was used as a carrier gas at a constant flow rate of 1 mL/min. The constituents were identified by comparing their mass spectra with NIST library files, and library matching and quality higher than 0.5% and 80% were used, respectively.

### 2.7. Total Phenolic Content

The assay method was conducted according to the protocol described by Luu et al. (2023) [17]. A 25 μL coffee sample was mixed with 50 μL 10% (*v*/*v*) Folin-Ciocalteu reagent and left for 5 min. Then 200 μL 7.5% sodium bicarbonate was added and kept in the dark at room temperature (25 °C) for 2 h. The samples were measured at a wavelength of 765 nm using a microplate reader. Results are expressed as gallic acid equivalent per volume of sample (GAE/mL).

### 2.8. Antioxidant Analysis

The antioxidant activities of coffee samples using cold and hot brew methods were determined using ferric ion reducing antioxidant (FRAP), 2,2-diphenyl-1-picrylhydrazyl (DPPH) radical scavenging, and oxygen radical absorbance capacity (ORAC) assays from previously reported methods [17]. Briefly, the FRAP assay utilized FRAP reagent, acetate buffer, and FeCl_3_.6H_2_O solution, and it was measured at an endpoint detection at 600 nm. The DPPH radical scavenging assay, on the other hand, used DPPH radical solution, and it was detected at a wavelength of 520 nm. ORAC assays use 2,2′-azobis(2-amidinopropane) dihydrochloride and sodium fluorescein as the main reagents, with kinetical detection at 485 nm excitation wavelength and 528 nm emission wavelength. The wavelength was visualized using a Synergy HT 96-well UV-visible microplate reader and Gen 5 data analysis software (BioTek Instruments, Inc., Winooski, VT, USA). Trolox solutions were used as the standards. Results were expressed as Trolox equivalent per volume sample (µmol TE/mL).

### 2.9. Statistical Analysis

All measurements were conducted in triplicate. The response surface using factorial and central composite designs was conducted using Design of Expert software v.13 (Minneapolis, MN, USA). The independent *t*-test was used to test the significant differences between cold brew and hot brew in terms of physicochemical and chemical characteristics.

## 3. Results and Discussion

### 3.1. Central Composite Design

The CCD design matrix was applied to all responses, and 30 randomized runs were generated by the software (Table 2) to avoid biases. Across these runs, the TDS ranged between 1.19% and 3.99%.

#### Effects of Brewing Conditions on Total Dissolved Solids (TDS)

The effects of water temperature, C2WR, coffee mesh size, and extraction time were assessed using the analysis of variance (ANOVA). A two-factor interaction (2FI) model was chosen, and the result is shown in Table 3. In this factorial design model, the 2FI model presented the best fitness of the model as determined by R^2^ and the highest “Adjusted and Predicted R^2^”. Therefore, only two-factor interaction terms were tested, not quadratic terms.

The model equation for calculating the total dissolved solid was achieved using the 2F1 design model. The empirical relationship between the response and the independent variables in the coded units based on the experimental results is given in Equation (7):

TDS = 2.46122 + 0.144815A + 1.0562B + −0.190463C + −3.9186e-17D + 0.0548958AB + 0.0123958AC + 0.0265625AD + −0.0548958BC + 0.0159375BD + 0.0159375CD (7)

The TDS of the CBC ranged between 1.19 and 3.99%. The highest TDS was measured with the following brewing conditions: water temperature of 30 °C, C2WR of 1:6, coffee mesh size of 0.25 mm, and extraction time of 24 h. The lowest TDS measurement was observed when the brewing conditions were at the opposite end of the brewing range, i.e., water temperature 4 °C, C2WR 1:16, coffee mesh size 0.71 mm, and extraction hour 7 h. ANOVA identified the parameters that significantly influenced the TDS of CBC, with the factors being A: water temperature, B: C2WR, and C: coffee mesh size, and confirmed that the overall model was highly significant. As for the pairwise interactions, only AB and BC were confirmed to significantly impact TDS.

**Figure 1:**
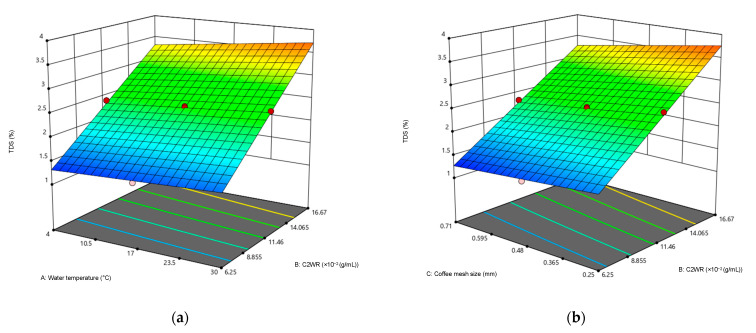
3D contour plots generated by Design Expert (Stat Ease, Inc.) of the significantly interacting model terms (**a**) A: water temperature and B: C2WR; (**b**) B: C2WR; and C: coffee mesh size. The dots indicated the axial and centre design points. The red zone showed high values of TDS obtained, whereas the lower TDS percentage were shown as the color that changed towards the blue zone.

A three-dimensional response surface plot was constructed to determine the optimal levels of each parameter to achieve the TDS of 1.35%, plotting the response (total dissolved solids, %) on the *z*-axis against any two independent parameters while maintaining other variables at their optimal values. These surface plots allowed the visualization of the optimum values for each parameter that yielded the desired response. Figure 1a shows the interaction between water temperature and C2WR. A combination of a higher water temperature (30 °C) and a higher C2WR (1:6 g/mL) led to the highest TDS reaching 3.99%, with the coffee mesh size and extraction hours maintaining at 0.48 mm and 15.5 h, respectively. Figure 1b illustrates the interaction between C2WR and coffee mesh size while keeping the other variables constant. It can be observed that the TDS of CBC increased as the C2WR increased while the coffee mesh size decreased, with the highest TDS measured at 3.99%, the C2WR at 1.6 g/mL, and the coffee mesh size at 0.25 mm.

The R^2^, adjusted R^2^, and predicted R^2^ values were calculated to be 0.9950, 0.9924, and 0.9897 with an adequate precision of 62.4579, showing an adequate signal to navigate the design space. The present model showed a LOF F-value of 1.18, implying that the lack of fit was not significant. Hence, the model could be used for the prediction.

### 3.2. Optimization and Validation of the Predicted Model for Brewing Conditions of Cold Brew Coffee

The optimal levels of each parameter were determined for the TDS of 1.35%, which was the TDS of coffee brewed using the French Press method. The Design Expert software generated the following conditions to achieve a TDS of 1.35% (Table 4).

Three experimental runs using the predicted optimized conditions were conducted to validate the RSM model. Table 5 displays the predicted mean, including a 95% prediction interval (PI), and the observed mean with standard deviation.

The test run showed that the model was significant, and the observed means fell within the 95% prediction interval. Thus, the model was validated and could be used for prediction.

In this study, water temperature, C2WR, and coffee mesh size significantly influenced the TDS of CBC. The TDS increased with the increase in extraction temperature from 4 to 30 °C. This can be attributed to the increased solubility of solutes. At a higher water temperature, water is able to extract solutes that are moderately water-soluble at lower temperatures [20]. Similar to our findings, Rey Castaneda-Rodriguez et al. (2020) reported a significantly higher total soluble solid content for coffee brewed at 25 °C than for coffee brewed at 10 °C [21]. Angeloni et al. (2019) also reported similar findings, showing that temperature significantly affected more total solids in coffee prepared at 22 °C compared with 5 °C [22].

When other brewing conditions (water temperature of 4 °C, coffee mesh size of 0.71 mm, extraction time of 7 h) were kept constant, it was found that the TDS increased from 1.19 to 3.06% as the C2WR increased. TDS refers to the amount of dissolved material mass in the beverage and can consist of volatile and non-volatile compounds [11]. A higher C2WR indicates that a higher amount of volatile and non-volatile compounds may naturally be present [23] and thus would produce a higher TDS.

Aside from that, coffee mesh size appeared to significantly influence the TDS of coffee. The TDS had an inverse relationship with the coffee mesh size. At a constant brewing condition (water temperature 17 °C, C2WR 11.46 × $10^{-2}$ g/mL, extraction time 15.5 h), the TDS measured for 0.25 mm and 0.71 mm coffee mesh sizes were 2.72% and 2.32%, respectively. This can be explained by the higher surface area of the coffee grind. A smaller coffee particle exhibits a larger surface area, resulting in more efficient extraction of solutes [24]. Other studies published different findings. For example, a study by Portela et al. (2021) reported that particle size exhibited different behaviors according to the different coffee species used, e.g., Arabica and Robusta. For Robusta coffee, total soluble solids extraction was higher in the finer grind, whereas Arabica coffee was reported otherwise [25]. The author concluded that the difference in coffee granular structure makes the extraction process distinctly different [25]. Contradictory to our findings, Cordoba et al. (2019) also reported that a coarser coffee grind presented a higher TDS in comparison to the finer coffee grinds. This situation was due to the indirect immersion method used by the author. The coffee grinds were placed in a filter bag prior to submerging in water, thus producing a more caking effect for medium grinds that would impede solute extraction [7].

In the brewing process, water temperature is considered the main driving force in extracting the chemical compounds present in the coffee. However, this is not the case for CBC, where the water temperature is at room temperature or lower. Thus, it needs to be compensated with a longer steeping time [7]. Cordoba et al. (2019) reported that contact time was statistically significant for TDS, e.g., higher TDS at 22 h of extraction [7].

In contrast, the present study revealed that extraction time does not significantly influence the TDS of coffee. This can be explained by the kinetic plots of compound extraction. Rey Castaneda-Rodriguez studied the extraction kinetics of CBC. It was reported that the initial TSS extraction increased over the first 3 h and slowed down to reach equilibrium at approximately 9 h. It is also noteworthy to say that there was no significant difference in TSS for a brewing time longer than 6 h [21]. This explains the result obtained in our study, which found no significant difference in TDS for all brewing at 7, 15.5, and 24 h.

### 3.3. Characterization of Cold and Hot Brew Coffee

#### 3.3.1. Physicochemical Comparison between Cold and Hot Brew Coffee

The physicochemical characteristics of CBC and hot brew coffee were compared, and the results are shown below (Table 6). The results showed that the TDS of CBC and its hot brew counterpart were comparable. Furthermore, the pH value obtained for both brews was the same. However, the cold brew had a significantly higher TA for all pH endpoints (pH 6.0, 8.0, and 8.2).

One of the objectives of this study was to optimize CBC close to a hot brew equivalent method, thus accounting for the similar TDS and pH readings. Nevertheless, to achieve a similar TDS from the cold brew technique, a higher C2WR was needed since a lower water temperature was used for brewing. Consequently, a higher C2WR would result in much more bioactive compounds being extracted. Although the amount of bioactive compounds could not be directly measured and reflected by TDS, TA could be measured and showed a higher value than their hot brew counterparts.

To the authors’ knowledge, this is the first study that optimizes, matches, and compares cold brew and hot brew at similar total dissolved solids. Other studies used the same C2WR for both hot and cold brew methods, which would logically result in significantly different properties. Angeloni et al. (2019) and Bilge (2020) used a 1:10 coffee-to-water ratio and found that TDS and pH were different between CBC and hot brew coffee [22,24]. The total solid was observed to be lower in cold extraction methods compared with its benchmark hot brew extraction [22]. The differences in water temperature influenced the pH of the final brewed coffee, whereby the pH of the final brew from cold brew was less acidic and ranged from 5.5 to 5.7 compared with the hot brew (French Press) at pH 5.2 [22]. Similarly, Bilge (2020) found that acidic compounds were extracted more in hot brew coffee, resulting in a lower pH of 5.0 compared with the pH of 5.2 in CBC [24]. Contrastingly, Fibrianto et al. (2018) reported that the pH of the final brew was not affected by the brewing method [26]. Additionally, Asiah et al. (2019) and Fuller and Rao (2017) also found that there was little difference in Brix and pH between CBC and hot brew coffee [4,27].

Even though similar pH readings were obtained from hot and cold brew coffee, the titratable acidity was higher in the hot brew coffee [16]. Similar results were also reported by other authors [7,28]. Interestingly, the CBC exhibited a higher TA than hot brew coffee in our study. In a study by Rao and Fuller (2018), the coffee variety, grind size, coffee roast profile, and C2WR remained the same; only the water temperature and brewing time were different [16]. Generally, a higher temperature would result in more extractable soluble acidic compounds, but a longer brewing time used in CBC may compensate for the low water temperature used. However, it should be noted that certain compounds are temperature-dependent, less sensitive to the extraction time, and thus more soluble at a higher temperature [4].

The TA results in our study can be explained by the difference in C2WR used. To achieve a similar TDS, a 1:14 coffee-to-water ratio was used for CBC, whereas a 1:18 C2WR was used for hot brew coffee. The acidic components would be more concentrated in CBC since the water used is less than that used in hot brew coffee.

#### 3.3.2. Total Phenolic Content and Antioxidant Activity Comparison between Cold and Hot Brew Coffee

The antioxidant capacities and total phenolic content of CBC were compared with those of its hot brew counterpart and are shown below (Table 7). The table above showed that the total phenolic content and antioxidant activities measured by FRAP and DPPH radical scavenging assays were similar between cold brew and hot brew coffee (*p* > 0.05). This was predicted because the method of CBC preparation was optimized to result in similar TDS obtained for hot brew coffee. However, the CBC exhibited significantly higher antioxidant activity than its hot counterpart, as measured by the ORAC assay.

The total phenolic content was measured using the Folin–Ciocalteu (FC) Assay, which is a colorimetric ET-based method. This assay utilizes the transfer of electrons from phenolic compounds to the FC reagent (a mixture of phosphor-molybdate and phosphotungstate) to form a blue-colored complex [29].

In plants, antioxidants work in two major pathways, i.e., hydrogen atom transfer (HAT) and single electron transfer (SET) [30]. While FRAP, TPC, and DPPH radical scavenging assays utilize the electron transfer reaction, the ORAC assay utilizes the HAT mechanism [31]. The ferric reducing antioxidant power (FRAP) assay is a colorimetric SET-based method. It measures the reduction of ferric ion (${Fe}^{3+}$) by antioxidants to form a blue-colored ferrous (${Fe}^{2+}$) complex. The intensity of the end product is then measured and quantified using a spectrophotometer to indicate the reducing power of the antioxidant tested [31].

2,2-diphenyl-1-picrylhydrazyl (DPPH) radical scavenging assay is also a colorimetric SET-based assay used to estimate the radical scavenging capacity of plants. The principle of this method utilizes the electron donation of antioxidants to neutralize the DPPH radical (purple in color), resulting in a color change that is measured by the spectrophotometer [31]. The oxygen radical absorbance capacity (ORAC) assay is a fluorometric HAT-based assay that measures the decrease in the fluorescence of a target under a constant flux of peroxyl radical [29,31].

The CBC samples showed that they had higher antioxidant activities according to the HAT-based mechanism than the hot brew, whereas the antioxidant activities according to the SET-based mechanism were not significant between the two samples. However, further investigations are needed to look into the cause of the difference in antioxidant activity from the ORAC Assay.

#### 3.3.3. Volatile Compounds in Cold and Hot Brew Coffee

The volatile compounds in cold brew and hot brew coffee samples were analyzed using GCMS, and the results are shown in Table 8. A total of 23 volatile compounds were identified in CBC samples, while in hot brew coffee samples, 16 volatile compounds were detected. The main compounds detected in both cold and hot brew samples were furans and pyrazines. Other minor volatiles detected were phenol, pyridine, ketone, aldehyde, oxime, and pyrrole. This agrees with a study by Cordoba et al. (2021), which found that furans and pyrazines are the major volatile compounds in coffee [32].

Pyrazine is associated with nutty, earthy, roasty, and green aromas, while furans are linked to sweet, caramel, fruity, roasted, and earthy aromas and flavors [32]. Our findings showed that the percentage area of volatile compounds was higher in CBC samples than in hot brew coffee. Apart from that, some volatile compounds, including 1-methyl-1H-pyrrole, Furan, 2-(methoxymethyl)-, Pyrazine, 2,5-dimethyl-, Pyrazine, 2,6-dimethyl-, Pyrazine, 2-ethyl-5-methyl-, Pyrazine, 3-ethyl-2,5-dimethyl-, Furfuryl formate, and 2-Methoxy-4-vinylphenol were identified in cold brew samples but not detected in the hot brew. Only 3(2H)-Furanone and dihydro-2-methyl- were detected in the hot brew but were not detected in CBC. This suggests that different extraction conditions play a role in volatile compound extraction.

#### 3.3.4. Phenolic Compounds in Cold and Hot Brew Coffee

The phenolic compounds were analyzed for both cold and hot brew samples. The phenolic coffee brewed using cold brew and hot brew (French Press) methods was analyzed by LC-ESI-MS/MS using twenty-four phenolic standards, including apigenin, caffeic acid, cinnamic acid, chlorogenic acid, p-coumaric acid, 3,4-dihydroxybenzoic acid, (-)-epigallocatechin gallate, ferulic acid, galangin, gallic acid, genistein, hesperidin, 4-hydroxybenzoic acid, kaempferol, isorhamnetin, luteolin, myricetin, naringenin, quercetin, rosmarinic acid, sinapic acid, rutin, syringic acid, and vanillic acid. However, all 24 phenolic compounds could not be detected in both coffee brews. This could be due to the fact that the amount of the compounds presented in the coffee brews is lower than the detection limit of the method used here.

#### 3.3.5. Caffeine Content in Cold and Hot Brew Coffee

Further analysis showed that caffeine was present in both samples (Table 9). The caffeine content was significantly higher in CBC than in its hot brew counterpart.

The amount of caffeine is significantly higher in CBC compared with hot brew coffee. This is similar to the findings by Fuller and Rao (2017), whereby cold brew samples had higher concentrations of caffeine, regardless of the roasting profile [4]. This is contradictory to the finding by Rao et al. (2020), where the caffeine concentration was similar in both cold and hot brew coffee samples [33].

#### 3.3.6. Acid Profiles of Cold and Hot Brew Coffee

Organic acids were analyzed using HPLC, and the results are tabulated as shown in Table 10. In general, CBC exhibited a much higher number of organic acids compared with hot brew coffee. L-malic acid was the main acid in both samples, while tartaric acid was not detected. The organic acids detected in samples below the LOQ were not quantified and were written as not detected (ND). L-malic, L-ascorbic, acetic, citric, fumaric, and succinic acids were significantly higher in cold brew samples compared with their hot counterparts. While oxalic and propionic acids were not detected in hot brew coffee, they were detected in small quantities in the cold brew samples.

## 4. Conclusions

The findings from this study showed that the brewing condition, water temperature, C2WR, and coffee mesh size influence the TDS of CBC. The optimized brewing conditions for CBC were water temperature (4 °C), C2WR (1:14), coffee mesh size (0.71 mm), and extraction time (24 h). The comparison between CBC and hot brew coffee samples with similar TDS revealed that there was no significant difference in total phenolic content or antioxidant activity as measured by the FRAP and DPPH radical scavenging assays. Even though the TDS and pH were similar for both samples, the titratable acidity was higher in CBC at all endpoints. The volatile compounds, caffeine content, and organic acid concentration were significantly higher in CBC than in the hot brew. In brief, CBC may not render higher health-beneficial properties than the hot brew, especially in terms of antioxidant activities and total phenolic content. However, the cold brew method can result in a difference in sensory properties and a higher caffeine content. The model for the prediction of TDS can also benefit food services or industries for the optimization of brewing conditions to obtain different characteristics of CBC.

## Figures and Tables

**Table 1:** Experimental values and levels of selected factors for central composite design.

Variables	Experimental Values		
	−1	0	1
Water temperature (°C)	4	17	30
Coffee-to-water ratio (g/mL)	6.25	11.46	16.67
Coffee mesh size (mm)	0.25	0.48	0.71
Extraction time (h)	7	15.5	24

**Table 2:** Experimental runs for CCD (TDS).

Experimental Run	Brewing Conditions				Response
	Water Temperature (°C)	Coffee-to-Water Ratio $\left(\times 10^{-2}\;g/mL\right)$	Coffee Mesh Size (mm)	Extraction Time (h)	TDS (%)
1	4	16.67	0.71	24	3.06
2	17	11.46	0.48	15.5	2.30
3	30	16.67	0.25	7	3.91
4	4	6.25	0.71	7	1.19
5	17	11.46	0.25	15.5	2.72
6	30	6.25	0.71	24	1.36
7	30	11.46	0.48	15.5	2.64
8	30	6.25	0.25	7	1.64
9	4	16.67	0.25	24	3.57
10	17	11.46	0.48	15.5	2.41
11	30	6.25	0.71	7	1.36
12	30	16.67	0.25	24	3.99
13	30	6.25	0.25	24	1.64
14	4	16.67	0.25	7	3.66
15	17	16.67	0.48	15.5	3.34
16	4	11.46	0.48	15.5	2.38
17	17	11.46	0.48	15.5	2.38
18	17	11.46	0.48	15.5	2.49
19	4	6.25	0.71	24	1.19
20	4	6.25	0.25	7	1.53
21	17	11.46	0.48	15.5	2.44
22	17	11.46	0.48	24	2.55
23	4	6.25	0.25	24	1.36
24	30	16.67	0.71	7	3.49
25	30	16.67	0.71	24	3.57
26	17	11.46	0.48	15.5	2.47
27	17	11.46	0.48	7	2.47
28	17	6.25	0.48	15.5	1.36
29	4	16.67	0.71	7	3.06
30	17	11.46	0.71	15.5	2.32

**Table 3:** Result of ANOVA for the 2FI model identifying factors and pairwise interactions significantly influencing TDS.

Source	Sum of Squares	DF	Mean Square	F Value	*p*-Value	
Model	21.23	10	2.120	377.52	<0.0001	***
A-water temperature	0.3775	1	0.3775	67.13	<0.0001	***
B-Coffee-to-water ratio	20.08	1	20.08	3570.88	<0.0001	***
C-coffee mesh size	0.6530	1	0.6530	116.12	<0.0001	***
D-extraction time	0.0000	1	0.0000	0.00	1.00	
AB	0.0482	1	0.0482	8.57	0.0086	**
AC	0.0025	1	0.0025	0.4372	0.5164	
AD	0.0113	1	0.0113	2.01	0.1727	
BC	0.0482	1	0.0482	8.57	0.0086	**
BD	0.0041	1	0.0041	0.7227	0.4058	
CD	0.0041	1	0.0041	0.7227	0.4058	
Residual	0.1068	19	0.0056			Not significant
Lack of fit	0.0821	14	0.0059	1.18	0.4594	
Pure error	0.0248	5	0.0050			
Standard deviation	0.0750			$R^{2}$		0.9950
Mean	2.46			Adjusted $R^{2}$		0.9924
C.V. (%)	3.05			Predicted $R^{2}$		0.9897
				Adequate precision		62.4579

** *p* < 0.01, and *** *p* < 0.001.

**Table 4:** Optimized brewing conditions generated by Design Expert software.

Brewing Conditions			
Water Temperature (°C)	Coffee-to-Water Ratio (g/mL)	Coffee Mesh Size (mm)	Extraction Time (h)
4	1:14	0.71	24

**Table 5:** Response surface methodology (RSM) model validation via predicted values vs. observed values.

Response	Predicted Mean	95% PI Low	Observed Mean	95% PI High	Std. Dev.
TDS *	1.31	1.16	1.37	1.45	0.07

* Significant model with insignificant lack of fit.

**Table 6:** Physicochemical characteristics of cold and hot brew coffee.

	Cold Brew Coffee	Hot Brew Coffee	*p*-Value
TDS (%)	1.28 ± 0.02	1.21 ± 0.02	0.0048 *
pH	5.45 ± 0.08	5.45 ± 0.03	0.9663
Titratable acidity, pH 6.0 (mL NaOH/20 mL coffee)	0.63 ± 0.02	0.41 ± 0.02	0.0096 *
Titratable acidity, pH 8.0 (mL NaOH/20 mL coffee)	1.99 ± 0.14	1.50 ± 0.02	0.00245 *
Titratable acidity, pH 8.20 (mL NaOH/20 mL coffee)	2.19 ± 0.12	1.67 ± 0.03	0.0115 *

* Statistically significant *p* < 0.05; results are the mean ± standard deviation from triplicate extractions.

**Table 7:** Total phenolic content and antioxidant activities of cold and hot brew coffee.

	Cold Brew Coffee	Hot Brew Coffee	*p*-Value
Total phenolic content (GAE/mL)	2.0281 ± 0.1538	2.0737 ± 0.1349	0.7193
Antioxidant assay			
FRAP assay (µmol TE/mL)	8.5374 ± 0.0916	8.6428 ± 0.4567	0.7305
DPPH radical scavenging assay (µmol TE/mL)	0.0066 ± 0.0002	0.0065 ± 0.0002	0.6005
ORAC assay (µmol TE/mL)	47.8460 ± 1.3109	34.8303 ± 3.7049	0.0171 *

* Statistically significant, *p* < 0.05; results are the mean ± standard deviation from triplicate extractions.

**Table 8:** Volatile compounds of cold and hot brew coffee.

Compound	Chemical Class	Cold Brew Coffee (%)	Hot Brew Coffee (%)	*p*-Value
1-methyl-1H-pyrrole	Pyrrole	0.26 ± 0.36	ND	
Pyridine	Pyridine	2.47 ± 3.49	1.24 ± 1.00	
Furan, 2-(methoxymethyl)-	Furan	0.28 ± 0.40	ND	
Pyrazine, methyl-	Pyrazine	1.90 ± 0.07	0.53 ± 0.75	
Pyrazine, 2,5-dimethyl-	Pyrazine	0.80 ± 0.01	ND	*
Pyrazine, 2,6-dimethyl-	Pyrazine	0.66 ± 0.06	ND	*
Pyrazine, ethyl-	Pyrazine	1.20 ± 0.08	0.58 ± 0.82	
Pyrazine, 2-ethyl-6-methyl-	Pyrazine	1.06 ± 0.03	0.38 ± 0.54	
Pyrazine, 2-ethyl-5-methyl-	Pyrazine	0.65 ± 0.00	ND	
Pyrazine, 3-ethyl-2,5-dimethyl-	Pyrazine	0.71 ± 0.02	ND	*
Furfural	Aldehyde	9.03 ± 0.08	4.18 ± 4.00	
Furfuryl formate	Aldehyde	1.67 ± 0.04	ND	*
Ethanone, 1-(2-furanyl)-	Furan	1.93 ± 0.06	0.72 ± 1.01	
2- furancarboxaldehyde, 5-methyl-	Furan	7.64 ± 0.08	3.28 ± 3.21	
2-furanmethanol, acetate	Furan	7.96 ± 0.11	2.34 ± 2.21	
Furan, 2,2′-methylenebis-	Furan	1.05 ± 0.03	0.52 ± 0.73	
1H-Pyrrole-2-carboxaldehyde, 1-methyl	Pyrrole	0.87 ± 0.07	0.36 ± 0.51	
2-Furanmethanol	Furan	6.56 ± 0.13	2.86 ± 2.79	
Oxime-, methoxy-phenyl-	Oxime	7.08 ± 0.11	8.30 ± 8.55	
1H-Pyrrole,1-(2-furanymethyl)-	Pyrrole	0.72 ± 0.07	0.40 ± 0.57	
Furan, 2-(2-furanylmethyl)-5-methyl-	Furan	0.26 ± 0.36	0.25 ± 0.35	
2-methoxy-4-vinylphenol	Phenol	0.28 ± 0.39	ND	
3(2H)-furanone, dihydro-2-methyl-	Ketone	ND	0.25 ± 0.35	

* Statistically significant *p* < 0.05; results are the mean ± standard deviation from duplicate extractions.

**Table 9:** Comparison of the caffeine content of cold and hot brew coffee with LC-ESI-MS/MS.

Compound (µg/g)	Cold Brew Coffee	Hot Brew Coffee	*p*-Value
Caffeine	40,807.61 ± 2742.61	29,710.21 ± 1228.60	0.00979 *

* Statistically significant *p* < 0.05; results are the mean ± standard deviation from triplicate extraction.

**Table 10:** Comparison of the organic acid profile of cold and hot brew coffee with HPLC.

Organic Acid	Cold Brew Coffee (mg/mL)	Hot Brew Coffee (mg/mL)	*p*-Value
Oxalic	0.2441 ± 0.0166	ND	0.0015
Tartaric	ND	ND	
Quinic	9.9074 ± 0.3222	8.9293 ± 1.4168	0.3541
Formic	6.3737 ± 0.3506	2.8997 ± 1.6198	0.0596
L-malic	225.0592 ± 2.5920	179.9038 ± 1.1090	0.0002
L-ascorbic	0.4428 ± 0.0067	0.3170 ±0.0204	0.0048
Acetic	15.1426 ± 0.2670	11.1902 ± 0.9038	0.0117
Citric	10.6322 ± 0.7767	6.5674 ± 0.5031	0.0029
Furmaric	0.0428 ± 0.0012	0.0297 ± 0.0029	0.0081
Succinic	28.4802 ± 4.3241	0.3638 ± 0.6301	0.0069
Propionic	0.4258 ± 0.0100	ND	0.0002

Statistically significant is considered at *p* < 0.05; results are the mean ± standard deviation from triplicate extraction.

## Data Availability

Data is contained within the article or supplementary material.

## Supplementary Materials

The following supporting information can be downloaded at: https://www.mdpi.com/article/10.3390/foods12122412/s1, Table S1: SRM transition and collision energies used in LC-ESI-MS/MS to detect caffeine; Table S2: chromatographic parameters, retention time, recovery of standard, concentration ranges and detection system of caffeine standard; Figure S1: high-performance liquid chromatograms of (A) organic acid standards, (B) cold brew coffee samples, and (C) hot brew coffee samples; Figure S2: liquid chromatography-electrospray ionization tandem mass spectrometry (LC-ESI_MS/MS) chromatograms of (A) phenolic standards of cold brew coffee and (B) hot brew coffee samples; Figure S3: LC-ESI-MS/MS chromatograms of caffeine standards at (A) concentration 0.625; (B) concentration 1.25; and caffeine chromatograms of (C) cold brew and (D) hot brew coffee samples.
