# Supplementary material for: Optimization of Cold Brew Coffee Using Central Composite Design and Its Properties Compared with Hot Brew Coffee
Source: Foods. 2023 Jun 19;12(12):2412. doi: 10.3390/foods12122412 (PMC10297883; doi:10.3390/foods12122412)

foods MDPI

Supplementary Materials

Optimization of Cold Brew Coffee Using Central Composite Design and Comparing with Hot Brew Coffee

Supplementary Table S1:

Compounds	Standards	Ion Mass	Parent Ions (*m*/*z*)	*SRM* Transitions (*m*/*z*) and Collision Energy (*V*)	*RF lens (V)*
1	Caffeine	[M+H]	195.188	110.143 (23.7 V), 122.982 (32.76 V), 138.214(19.91V)	181

Supplementary Table S2:

Compounds	Standards	Retention time (min)	Linear range (µg/mL)	Linear regression equation	Correlation coefficient (R^2)	LOD (µg/mL)	LOQ (µg/mL)	%RSD (Inter-day)	%Recovery		
									Low level (µg/mL)	Medium level (µg/mL)	High level (µg/mL)
1	Caffeine	1.175	0.0195–10	y =10373 x + 985.93	0.9978	0.10	0.35	0.35	93.35	100.52	106.66

Supplementary Figure S1:

The high-performance liquid chromatograms of (A) organic acid standards, (B) cold brew coffee sample and (C) hot brew coffee sample.

(A)

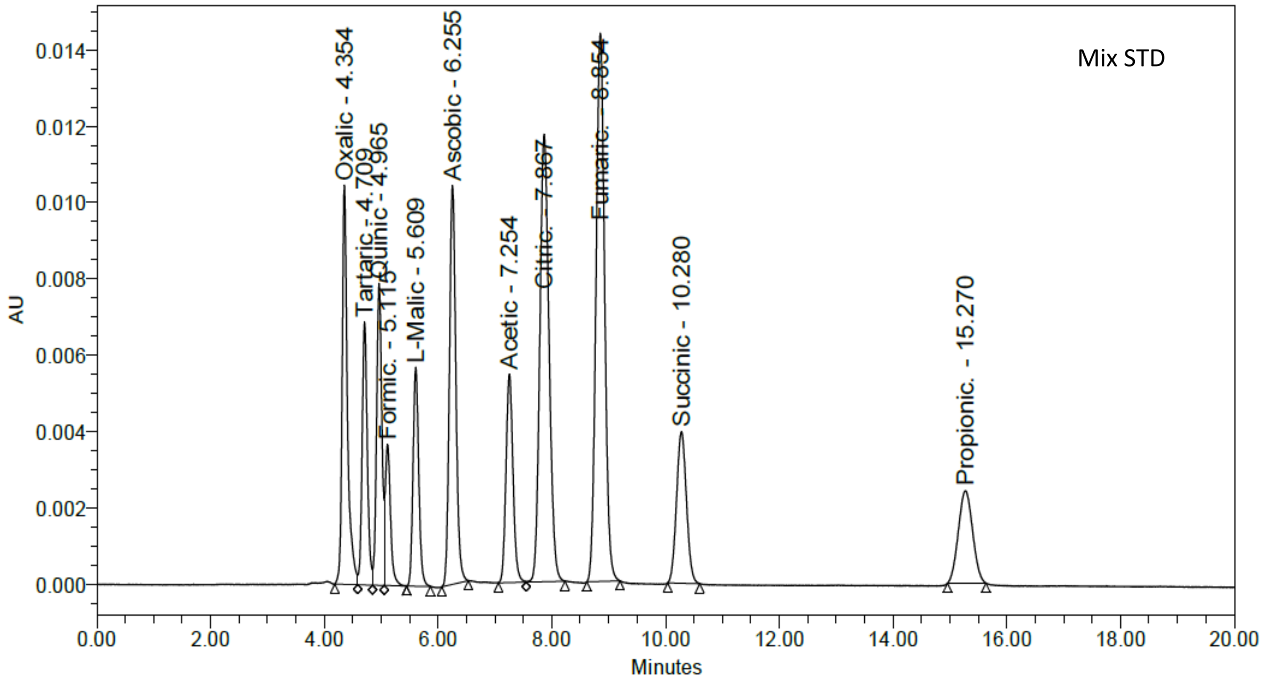

(B)

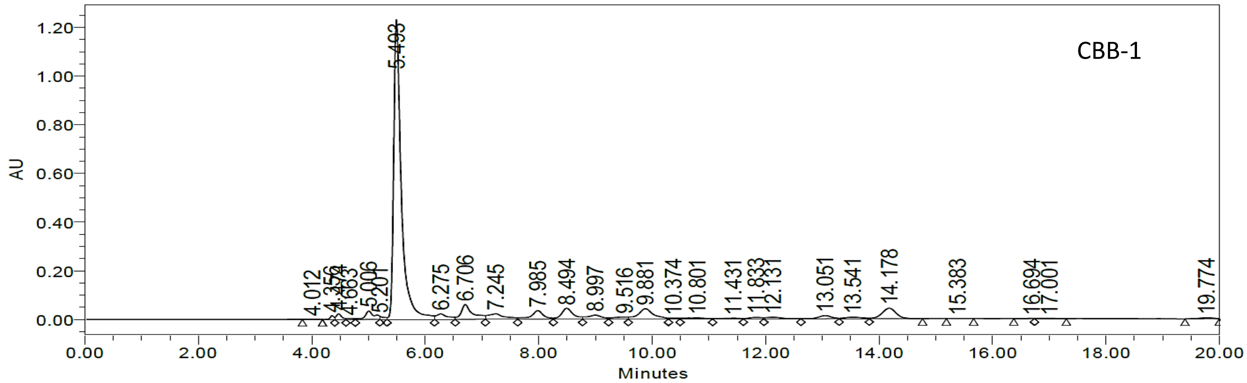

(C)

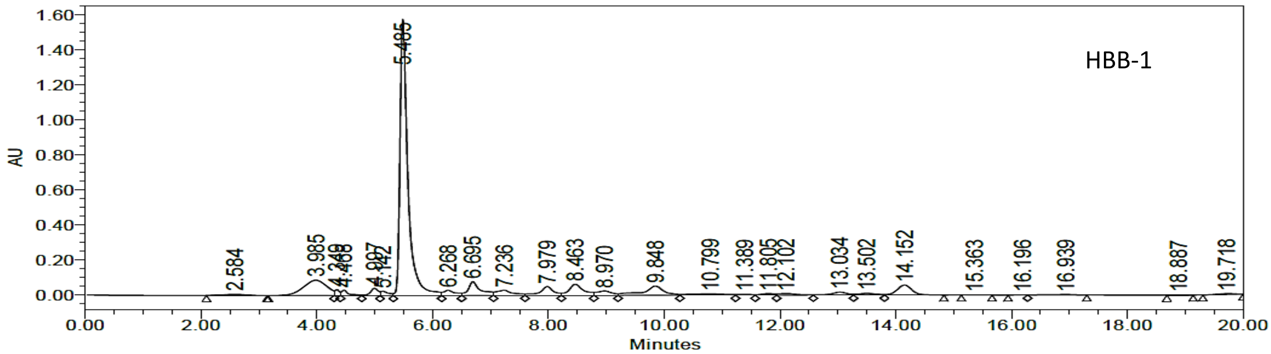

Supplementary Figure S2:

The liquid chromatography-electrospray ionization tandem mass spectrometry (LC-ESI-MS/MS) chromatograms of (A) phenolic standards of cold brew coffee and (B) hot brew coffee samples.

(A)

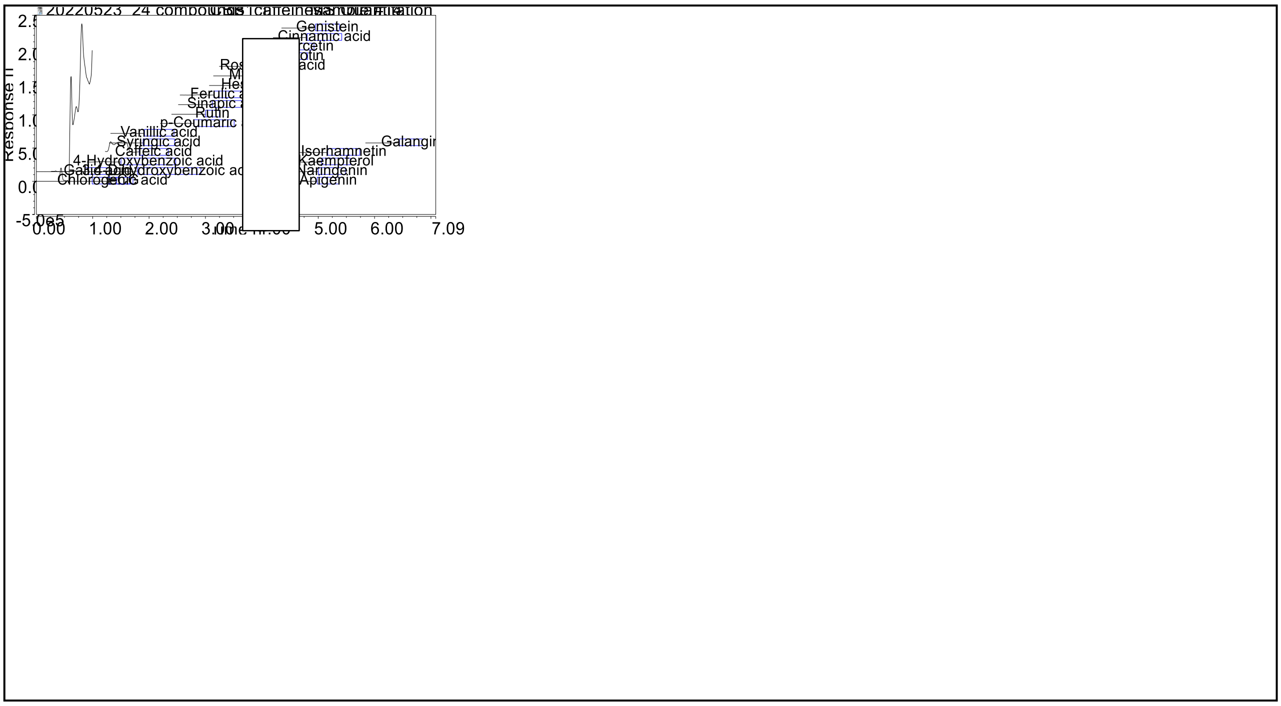

(B)

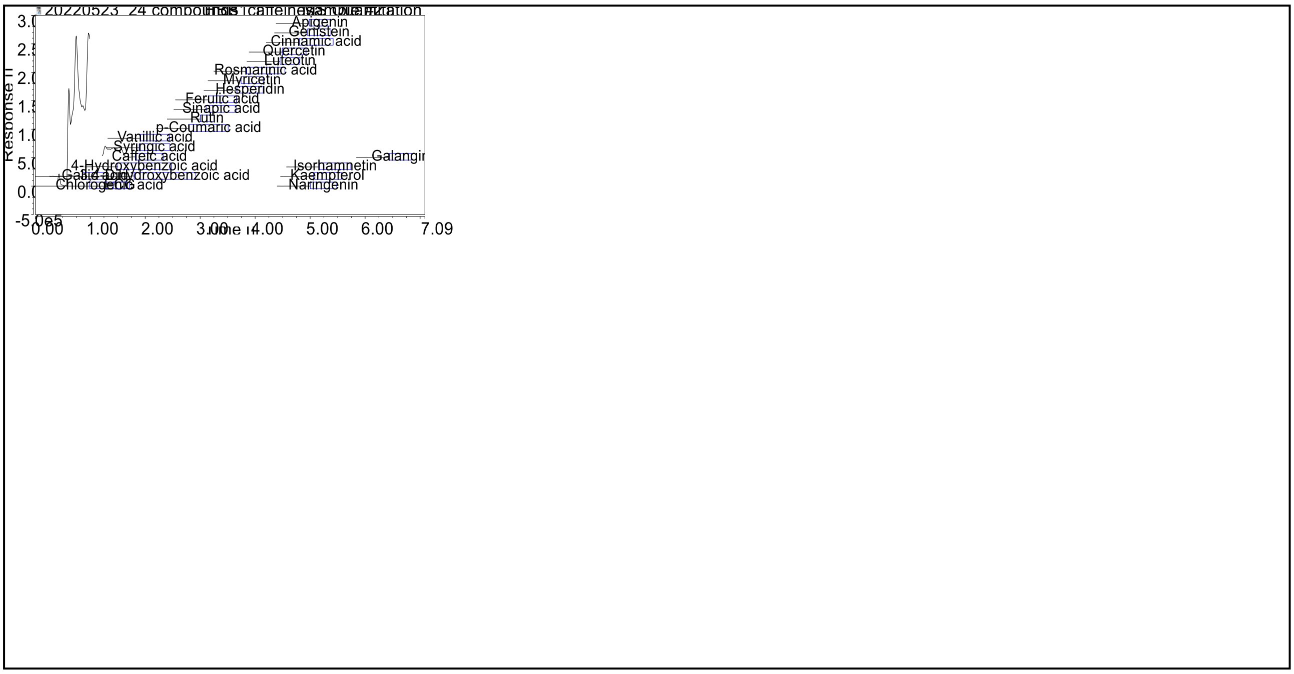

Supplementary Figure S3:

The liquid chromatography-electrospray ionization tandem mass spectrometry (LC-ESI-MS/MS) chromatograms of caffeine standards at (A) concentration of 0.625, (B) concentration of 1.25 and caffeine chromatograms of (C) cold brew and (D) hot brew coffee samples.

(A)

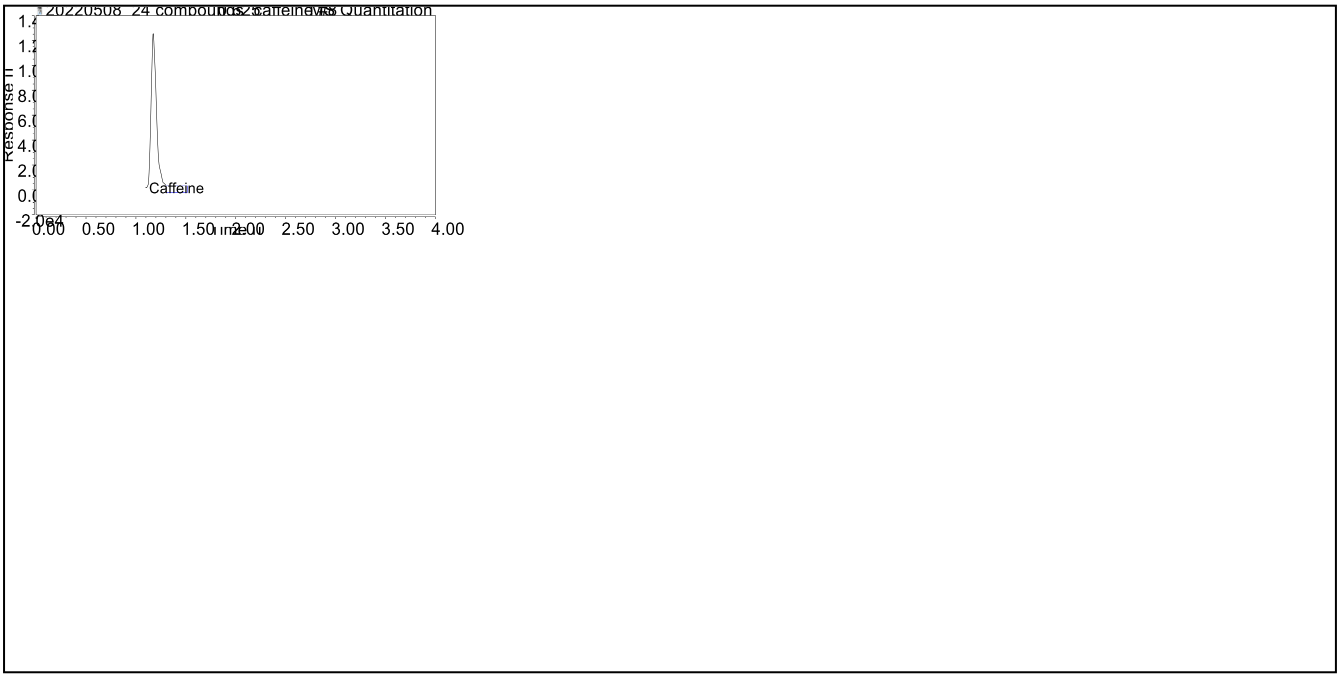

(B)

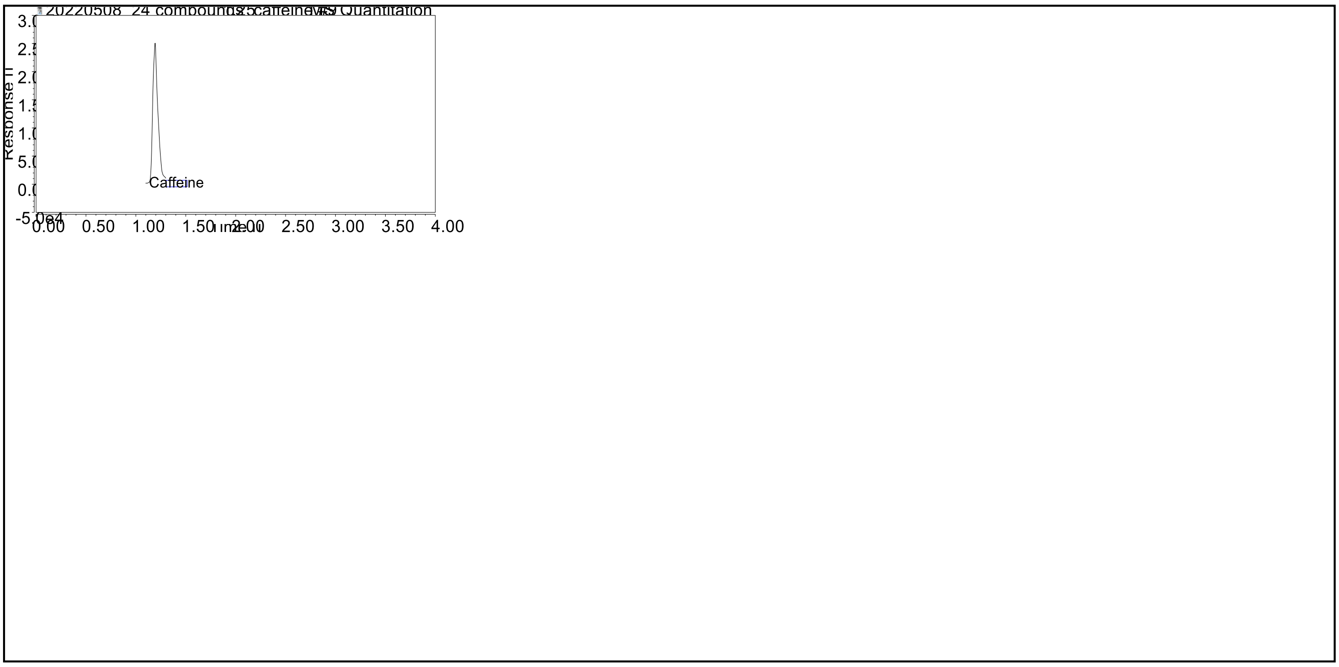

Supplementary Figure S3 (Cont.) :

The liquid chromatography-electrospray ionization tandem mass spectrometry (LC-ESI-MS/MS) chromatograms of caffeine standards at (A) concentration of 0.625, (B) concentration of 1.25 and caffeine chromatograms of (C) cold brew and (D) hot brew coffee samples.

(C)

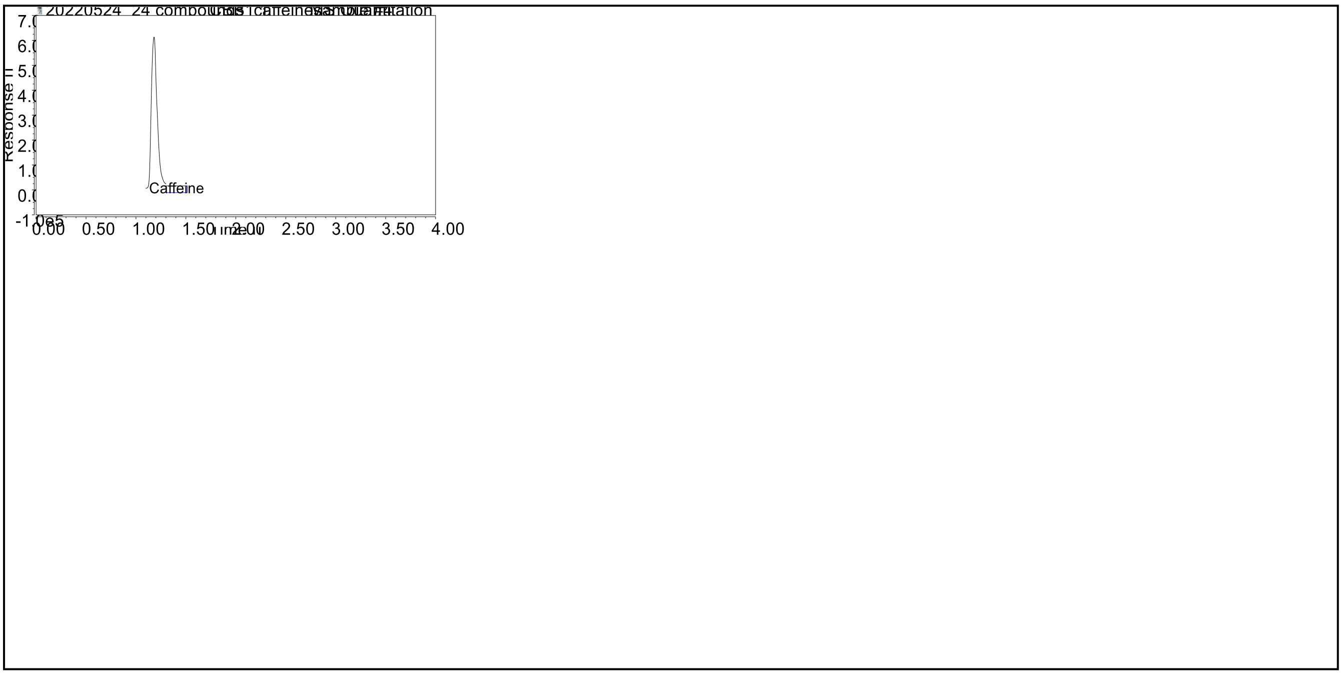

(D)

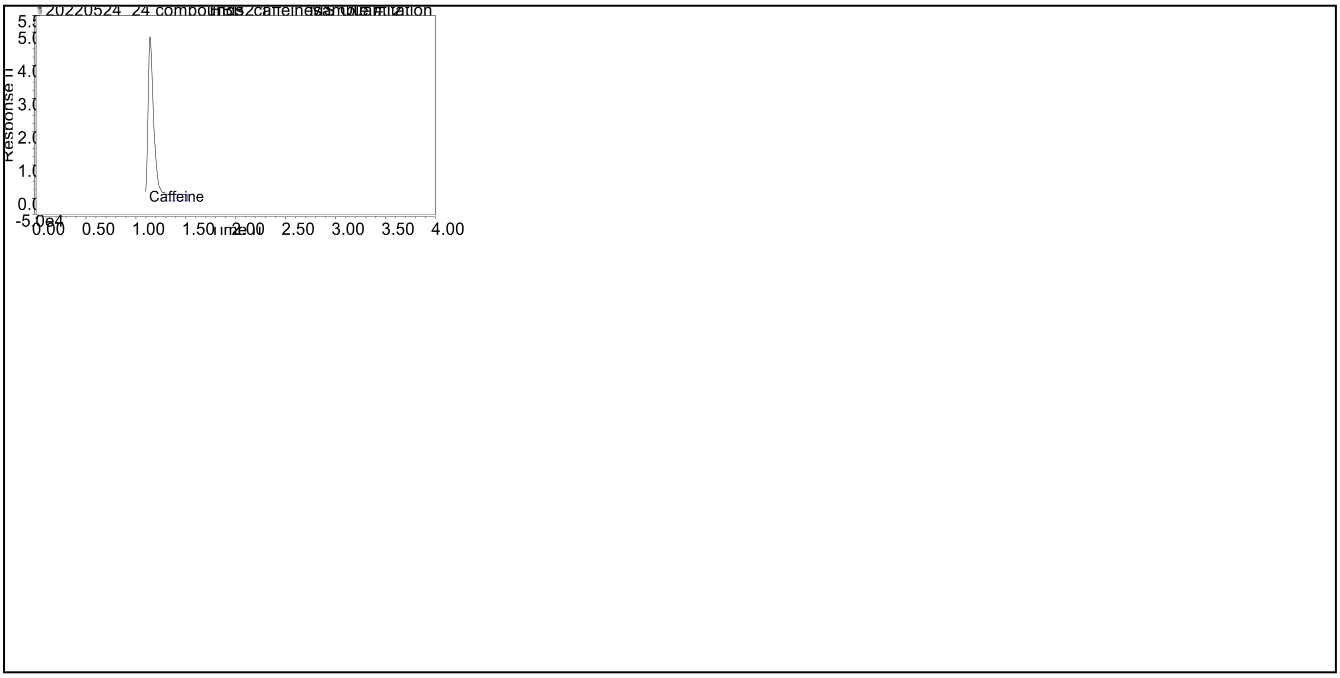

Supplement: Supplementary file 1 [file foods-12-02412-s001.zip › foods-2438873-supplementary.pdf]
